# Addressing the Gap: Mock Tribunal Teaching

**DOI:** 10.1192/bjo.2026.11328

**Published:** 2026-06-30

**Authors:** Catrin Comeau, Olaide Oladosu, Rakesh Puli

**Affiliations:** NHS, Cardiff, United Kingdom

## Abstract

**Aims:**

This project aimed to improve trainees’ understanding and effectiveness at Mental Health Act tribunals (MHTs) through education. It explored how training influences trainees’ understanding of the legal process, confidence in preparation and ability to present evidence.

MHTs are vital in the protection of detained patients’ rights. Despite this, many trainees in Wales lack the opportunity to observe them. This can lead to impaired effectiveness at tribunal and anxiety for many trainees.

**Methods:**

A session was designed comprising of a lecture introducing format and criteria of the tribunal, followed by a mock session. The mock was observed and was followed by a facilitated discussion.

Pre- and post-training questionnaires assessed knowledge of detention criteria, tribunal proceedings, confidence levels and interest in future educational sessions. Quantitative and qualitative data were obtained with Likert scales assessing interest in future educational sessions.

**Results:**

The key points from the pre-training survey (N=12) were a good awareness of why tribunals happen (12/12), some awareness of the legal criteria (5/12) and limited experience of taking part in tribunals (3 observed, 2 wrote reports, 1 oral evidence). All agreed that training would be helpful, and most were willing to take part.

Post-training survey (N=7) showed improvement in knowledge of detention criteria (6/7).Most respondents agreed that the session felt realistic and thought that the session would be helpful in the future.

**Conclusion:**

The improvement of trainee’s knowledge of detention criteria shows high educational impact and the positive feedback on the realism of the mock suggests that it should have high validity to real life. We plan to expand the session to offer it to more trainees and ultimately aim to open it for all professionals involved in MHTs.

